# Aortic valve replacement in octogenarians

**DOI:** 10.1186/1749-8090-2-33

**Published:** 2007-07-13

**Authors:** Amal K Bose, James D Aitchison, John H Dark

**Affiliations:** 1 Department of Cardiothoracic Surgery, The Freeman Hospital, High Heaton, Newcastle-upon-Tyne, NE7 7DN, UK

## Abstract

**Background and Aims:**

As our population ages and life expectancy increases the number of people aged over 80 and more referred for cardiac surgery is growing. This study sought to identify the outcome of aortic valve replacement (AVR) in octogenarians.

**Methods:**

68 patients aged 80 years or more underwent AVR at the Freeman Hospital, between April 2001 and April 2004. A retrospective review of the notes and outcomes from the patients' GP and the NHS strategic tracking service was performed. 54% (37) underwent isolated AVR whilst 46% (31) underwent combined AVR and CABG.

**Results:**

Follow up was 100% complete. The mean age was 83.1 ± s.d. 2.9 years, a mean gradient of 83 ± s.d. 31 mmHg and mean AVA of 0.56 cm^2^. The mean additive EuroSCORE was 8.6 ± s.d. 1.2, the logistic EuroSCORE mean 12.0 ± s.d. 5.9. In hospital 30 day mortality was 13 %. Survival was 80% at 1 year and 78% at 2 years. Median follow up was for 712 days. Stepwise logistic regression identified chronic obstructive airways disease as an independent predictor of mortality (p < 0.05). Survival was not adversely affected by the addition of coronary artery bypass grafts to aortic valve replacement, the presence of peripheral vascular disease, hypertension or diabetes. In this study duration of cross clamp or bypass time were not found to reach significance as independent predictors of mortality.

**Conclusion:**

Our study demonstrates that the operative mortality for AVR in the over eighties is good, whilst the mid to long term outcome is excellent There is a very low attrition rate with those undergoing the procedure living as long than their age matched population. This study confirms AVR is a safe, acceptable treatment for octogenarians with excellent mid term outcomes.

## Background

Life expectancy for both men and women has continued to rise in the UK. Data from 2002 shows life expectancy at birth for females born in the UK was 81 years, compared with 76 years for males. This contrasts with 49 and 45 years respectively at the turn of the last century. The expectation of life for people reaching the age of 80 has also increased and is now 7 years for men, 9 years for women in the U.K. (OPCS data). Cardiovascular disease is the largest cause of death in this age group. As our population ages, the number of people aged 80 or over referred for cardiac surgery is increasing with a particular rise in those with aortic valve disease. There is evidence that early outcomes in heart valve surgery are improving over the last decade [1] Previous studies have demonstrated good outcomes in terms of both operative mortality [2] and quality of life [3]. Age has also been shown to influence the decision to refer patients with aortic stenosis for surgery [4] with adverse outcomes [5]. This study sought to identify the medium term outcome of aortic valve replacement in octogenarians in a more recent setting.

## Methods

Between April 2001 and April 2004 all patients aged 80 years or more who underwent aortic valve replacement (AVR) or AVR and coronary artery bypass grafts at a single tertiary referral hospital in Northern England (Freeman Hospital) were identified. The notes were retrospectively reviewed. The patients' general practices were contacted to obtain follow up data, together with the hospital PATS database and the NHS strategic tracking service. Patients undergoing double valve replacement were excluded.

## Results

Sixty eight patients were identified. Data collection and follow up were 100% complete. The mean age was 83.2 ± s.d. 2.9 years, a mean gradient of 83 ± s.d. 31 mmHg and mean AVA of 0.56 ± s.d. 0.24 cm^2^. Fifty four percent (37) underwent isolated AVR whilst 46% underwent combined AVR and CABG. (Table 1) All the patients had bio-prostheses implanted. Two patients received stentless valves. All but one patient underwent first time valve replacement. One patient required root enlargement to accommodate a size 19 prosthesis.

The mean additive EuroSCORE was 8.6 ± s.d. 1.2, the mean logistic EuroSCORE was 12.0 ± s.d. 5.9, the mean Parsonnet score was 30.4 ± s.d. 4.3. In hospital 7 day and 30 day mortality were 4.4% and 13% respectively. Isolated AVR mortality was 10% at 30 days. Two patients (3%) were affected by a CVA or TIA. Atrial fibrillation occurred in 18 (26%), whilst seven patients required renal replacement therapy as a new intervention postoperatively in the form of continuous veno-venous haemofiltration. Mean hospital stay was 15 ± s.d. 12 and median 11 days (range 5 to 60 days).  See table 2.

**Figure 1:**
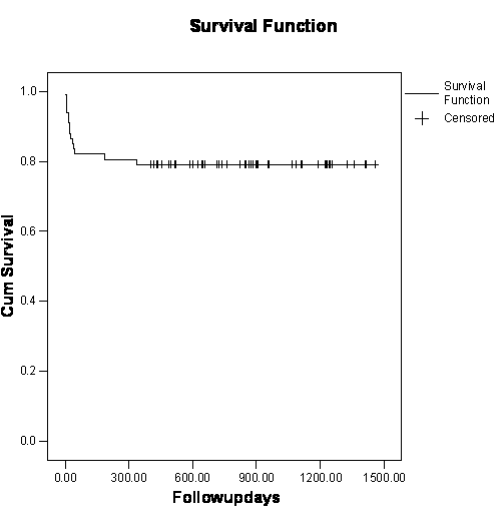
Survival curve for octogenarian aortic valve replacement.

Survival was 80% at 1 year and 78% at 2 years, see Figure 1. Median follow up was for 712 days. Stepwise logistic regression identified COAD as an independent predictor of mortality (p < 0.05). Survival was not adversely affected by the addition of coronary artery bypass grafts to aortic valve replacement, the presence of peripheral vascular disease, hypertension or diabetes. In this study, duration of cross clamp or bypass time were not found to reach significance as independent predictors of mortality.

## Comment

The current demographic trend throughout the developed world is for an ageing population with improved life expectancy. Data from the Society of Cardiothoracic Surgeons of Great Britain and Ireland national audit 2003 shows the average age of patients in the UK undergoing combined AVR and CABG has risen from 68 to just under 72 with a similar trend for isolated AVR [6].

Aortic valve replacement has been shown to be the most common valve surgery performed in this age group [7]. The simple additive Euroscore significantly under-predicted 30 day mortality in this sub-group of the general cardiothoracic patient population, with a mean Euroscore of 8.3. The mean logistic Euroscore was 12.0, which was closer to the actual mortality in this study. Previous studies have suggested that coronary artery bypass grafting combined with aortic valve replacement does not increase post operative risk [8], which is supported by our results.

Our study demonstrates that the operative mortality for AVR in the over eighties is good, whilst the mid to long term outcome is excellent. There is a very low attrition rate with those undergoing the procedure living as long as their age matched population. This study confirms AVR is a safe, acceptable treatment for selected octogenarians with excellent mid term outcomes. A surgical opinion should not therefore be withheld on the basis of age.

## Limitations

This is a small retrospective study which purely looked at hospital morbidity and mortality. Follow up mortality data was collected but there was no assessment of quality of life or symptom status in this data. A selection bias has not been excluded in this group proceeding to surgery. Perhaps because they were so carefully selected median survival was excellent.

## Competing interests

The author(s) declare that they have no competing interests.

## Authors' contributions

JD initiated the project. AB contributed to data collection and was primary author. JA performed the statistical analysis. All authors read and approved the final manuscript.

**Table 1:** Demographics, symptoms, risk factors

Variable (unit)	n (percentage)
Male	38 (58%)
Mean Age ± s.d. (years)	83.2 ± 2.9
NYHA I	7 (11%)
II	13 (21%)
III	29 (46%)
IV	14 (22%)
CCS 0	26 (42%)
I	8 (13%)
II	11 (18%)
III	14 (23%)
IV	3 (5%)
Impaired LV	11 (18%)
NIDDM	3 (4%)
Hypertension	29 (43%)
Renal Impairment/Failure	6 (9%)
COPD	12 (18%)
PVD	5 (8%)
Emergent/Urgent	18 (26%)

CCS – Canadian Cardiovascular Society

COPD – Chronic obstructive pulmonary disease

NYHA – New York Heart Association

LV – Left ventricle

NIDDM – Non insulin dependent diabetes mellitus

PVD – Peripheral vascular disease

**Table 2:** Post operative complications

Complication	n (percentage)
Atrial Fibrillation	18 (26%)
Renal Support (new CVVH)	7 (10%)
Respiratory Failure	12 (18%)
Bleeding requiring reopening	3 (4%)
CVA	1 (1%)
TIA	1 (1%)

CVA – Cerebro-vascular Accident

TIA – Transient Ischaemic Attack
